# The challenges and molecular approaches surrounding interleukin-2-based therapeutics in cancer

**DOI:** 10.1016/j.cytox.2018.100001

**Published:** 2018-12-10

**Authors:** Anthony Tang, Fiona Harding

**Affiliations:** AbbVie Biotherapeutics, 1500 Seaport Boulevard, Redwood City, CA 94063, United States

**Keywords:** Interleukin-2, Biologics, Melanoma, Renal cell carcinoma

## Abstract

Interleukin-2 has had a long history as a promising cancer therapeutic, being capable of eliciting complete and durable remissions in patients with metastatic renal cell carcinoma and metastatic melanoma. Despite high toxicity and efficacy limited to only certain patient subpopulations and cancer types, the prospective use of novel, engineered IL2 formats in combination with the presently expanding repertoire of immuno-oncological targets remains very encouraging. This is possible due to the significant research efforts in the IL2 field that have yielded critical structural and biological insights that have made IL2 more effective and more broadly applicable in the clinic. In this review, we discuss some of the molecular approaches that have been used to further improve IL2 therapy for cancer.

## Highlights

• IL2-based cancer therapies are limited by their toxicity and pleiotropy.
• Current engineering approaches target IL2 half-life and cell/receptor specificity.
• IL2 may enhance the efficacy of checkpoint inhibitors and CAR-T-based therapies.

## 1. Interleukin-2

Interleukin-2 (IL2) is a 15.5 kDa type I cytokine encoded on the long arm of chromosome 4 in humans [1]. It is composed of four distinct alpha helices following an up-up-down-down configuration [2] with other notable structural features including O-glycosylation on an N-terminal threonine and stabilization via a functionally critical disulfide bond between C58 and C105 of the polypeptide chain [3], [4], [5], [6], [7], [8]. Initially described in 1976 as a T cell growth factor in supernatants from mitogen-stimulated normal human peripheral blood cells [9], [10], [11], [12], IL2 has since been established as a critical component for the preservation of T cell homeostasis and proper immune regulation [13], [14]. Following demonstration of efficacy in clinical trials, high-dose IL2 was approved by the FDA for use in metastatic renal cell carcinoma (1992) and metastatic melanoma (1998) [15]. Although limited to only a small subset of patients, IL2 was capable of inducing complete, long-term remissions and was well-established for the treatment of these malignancies prior to the advent of targeted therapies (eg. BRAF and MEK inhibitors) and checkpoint inhibitors (eg. anti-PD1, anti-CTLA4). Despite the superior response rates and lower toxicity of these new therapies, the combined use of IL2 either in sequence [16] or in combination with targeted therapies [17] to establish optimal long-term responses remains a promising approach.

IL2 is mainly produced by CD4+ T cells following TCR activation and CD28 co-stimulation [18], [19]. Upon secretion, it can function in an autocrine/paracrine manner to upregulate its own receptor components in an initial positive feedback loop [20], [21], [22], [23]. CD8+ T cells, NK cells, and dendritic cells can also produce IL2 in more limited quantities [14]. IL2 has many important functions in lymphocytes, including the induction of proliferation, both cell survival and apoptosis (via activation-induced cell death), as well as the upregulation of cytotoxic cell activity [14]. These functions are primarily mediated by the activation of the JAK1 and JAK3 tyrosine kinases upon ligand binding and subsequent receptor heterodimerzation [24], [25]. This leads to downstream transcriptional events primarily mediated by a number of STAT transcription factors but also the activation of the MAPK [26], [27] and PI3K pathways [28], [29].

IL2 signals through a heterotrimeric receptor complex composed of distinct α, β, and γ chains (also known as CD25, CD122, and CD132, respectively). CD132 is used universally by every IL2 family cytokine (consisting of IL2, 4, 7, 9, 15, and 21) and upon cytokine binding, heterodimerizes with its uniquely corresponding signaling receptor chain (eg. IL4Rα (CD124), IL7Rα (CD127), IL21Rα (CD360), etc.) [30]. IL2 and IL15 are an exception to this, where despite a relative lack of amino acid sequence homology, both bind and signal through CD122 [31], [32]. Although all other IL2 family members signal through heterodimeric receptor complexes, IL2 and IL15 are additionally unique in that they can bind a third receptor component known as the α chain (also called CD25 and CD215 for IL2 and IL15, respectively). CD25 is not generally thought to directly participate in signal transduction, but instead, plays an important role in the presentation of the IL2 (or IL15) molecule via its interacting “sushi” domains - motifs compromised of 4 conserved cysteines that are typically involved in protein-protein interactions. This interaction increases the overall affinity of the cytokine for the intermediate affinity receptor (CD122/CD132 heterodimer) from the low nanomolar range into the picomolar range (high affinity CD25/CD122/CD132 heterotrimer) [14]. After receptor engagement and internalization, CD25 and CD215 may subsequently recycle to the cell surface, bypassing degradation unlike CD122/CD132 [33], [34]. This may allow for the CD25-mediated accumulation of an IL2 “reservoir” at the cell surface, resulting in prolonged cellular stimulation [35], [36]. This has been previously described for IL15/CD215 [37] and may be significantly less effective for IL2 due to the lower affinity of IL2 for CD25 when compared to the high affinity IL15 has for CD215 [36], [38]. Although typically thought to function in cis, CD25 has also been noted to function in trans by presenting IL2 to neighboring cells, which may be important in antigen presenting cells such as dendritic cells [39]. This is in contrast to CD215, which is thought to primarily function by presenting IL15 on the cell surface in trans [38], but also has functionality in cis [40]. The importance of the α chain in determining functional output is apparent in the comparison of IL2 versus IL15: two cytokines that both signal through the CD122/CD132 heterodimer but may cause noticeably different responses [41]. This is despite nearly identical ligand-receptor geometries and RNAseq gene expression profiles in the presence of saturating levels of cytokine. This suggests that differences observed between these two cytokines are instead mediated by expression of their alpha chains on different cell types and their roles in cis vs trans presentation [31].

Unlike other CD4+ and CD8+ T cells, T regulatory cells (T regs) are unable to produce IL2 but critically depend upon it for survival and function [42]. T regs readily respond to IL2 by virtue of high expression of all three IL2R chains. Experimental and mathematical models predict that T regs are capable of outcompeting T helper cells in close proximity for IL2 until a certain threshold of autocrine IL-2 secretion is attained, after which autocrine IL2 can act to upregulate CD25 on T helper cells. As such, out-competition of IL2 is a proposed mechanism through which T reg-mediated suppression may occur [23]. The importance of IL2 to T regs is further supported by evidence from IL2, CD25, or CD122-deficient mice, where the dominant phenotype is not immunodeficiency, but rather autoimmunity [43], [44], [45]. This underscores the interesting duality of IL2 to act as both immune suppressor and immune activator. In a similar fashion, differing sensitivities can also exist among other IL2-responsive cell types, where increased expression of CD122 by CD8+ T and NK cells governs their higher IL-2 sensitivity as compared to CD4+ T cells [46]. Ultimately, these differences in receptor expression can help to explain the differential quality of immune responses elicited by high vs low dose IL2 administration.

## 2. Challenges facing IL2 therapy

High-dose (600,000–720,000 IU/kg) IL2 monotherapy is capable of achieving complete responses in patients with metastatic renal cell carcinoma and metastatic melanoma, demonstrating objective response rates (ORRs) of approximately 20% and 16% in clinical trials [47], [48]. However, several drawbacks intrinsic to IL2 hamper its efficacy and broader application including its high toxicity/poor therapeutic index, ability to induce immunosuppressive responses through regulatory T cell (T reg) expansion, as well as its short circulatory half-life.

Perhaps the most restrictive issue with IL2 therapy is its dose-limiting toxicity. This can manifest in a number of ways including hypotension, vascular leak syndrome and edema, cardiac arrhythmias, as well as additional hematological and renal toxicities, which require proper management [49]. Approaches to addressing toxicity can be thought of in two complementary ways: (1) changing IL2 to make it into a less toxic molecule to allow for higher dosing, or (2) changing IL2 to make it more effective and allow for lower dosing. Clinical strategies attempting to address toxicity have involved changes in the dose and administration route. Lower doses (72,000 U/kg) were indeed more tolerable, but had reduced efficacy when compared to the high dose intravenous (iv) regimen [50]. Altering the route of IL2 administration can also significantly impact toxicity: iv bolus dosing results in peak serum concentrations but rapid clearance, while subcutaneous (sc) or intramuscular dosing results in significantly lower serum concentrations (∼2%) but a more sustained exposure [51]. Although reports are somewhat mixed, sc administration of IL2 whether alone or in combination with interferon alpha (IFNα) may yield reduced toxicity but may also have less activity compared to high dose iv IL2 [50], [52], [53], [54]. Intralesional dosing of injectable melanoma metastases appears promising, demonstrating superior efficacy to all other routes of administration with complete responses in over 60% of patients in addition to significantly reduced toxicity. However, this method may be somewhat limited by the laborious dosing regimen required at metastatic lesions, as activity is only confined to sites of treatment and does not appear to confer benefit to surrounding, untreated sites [55]. Similarly, attempts to increase the efficacy of IL2 by introducing combinations with other drugs have been undertaken to potentially reduce the amount of IL2 that needs to be administered. This has included combinations with other cytokines like IFNα, or chemotherapies, like 5-fluorouracil, to bolster responses while decreasing IL2 administration [56]. These combinations have not provided conclusive overall benefit over established IL2 strategies [56], [57], [58], [59], [60].

Another important limitation of IL2 therapy is its conflicting nature as a promoter of both immunosuppression via T regs, and immune activation via other CD4+, CD8+ T, and NK cells. This outcome is ultimately dependent on the administered dose of IL2, whereby high doses are immunostimulatory, but low doses are found to be immunosuppressive [61]. This creates a situation in which low doses could be detrimental to anti-tumor efficacy, while high doses are required for anti-tumor activity but also produce high toxicity, ultimately resulting in a more limited therapeutic index. In fact, low-dose IL2 administration is currently being pursued for the treatment of a number of autoimmune diseases including lupus and diabetes as well as for use in graft versus host disease [61]. Strategies to generate modified forms of IL2 such that it functions in a single capacity (either immunosuppression or immunoactivation) should subsequently help to improve its efficacy in either application and potentially increase its therapeutic index.

The major route of IL2 clearance is thought to occur through the kidneys [51] as it is a low molecular weight protein of 15.5 kDa, falling well below the glomerular filtration limit cited to be around 70 kDa [62]. The serum half-life of IL2 is very short - in the order of minutes to hours [51], [63], [64] - and consequently requires a rigorous dosing regimen comprised of administration every 8 h, for up to 14–15 doses [49] in order to sustain the high IL2 concentrations needed for antitumor activity. Optimization of the pharmacokinetic properties of protein therapeutics can lead to improved efficacy, cost, and convenience for patients and have been previously demonstrated for cytokines including IFNα, human growth hormone, and granulocyte-colony stimulating factor. Protein half-life extension can typically be achieved in a few different ways: modification of overall charge, increasing the hydrodynamic radius/molecular weight, and fusion to protein domains that mediate an extended serum half-life [62].

Many strategies have been attempted at the level of the clinic to mitigate the limitations of IL2, including changes in dosing route and combinations with other drugs. However, the issues of toxicity and efficacy still remain and molecular approaches modifying IL2 should be helpful in further improving IL2 in these areas.

## 3. IL2 engineering strategies

### 3.1. IL2 muteins

**Fig. 1:**
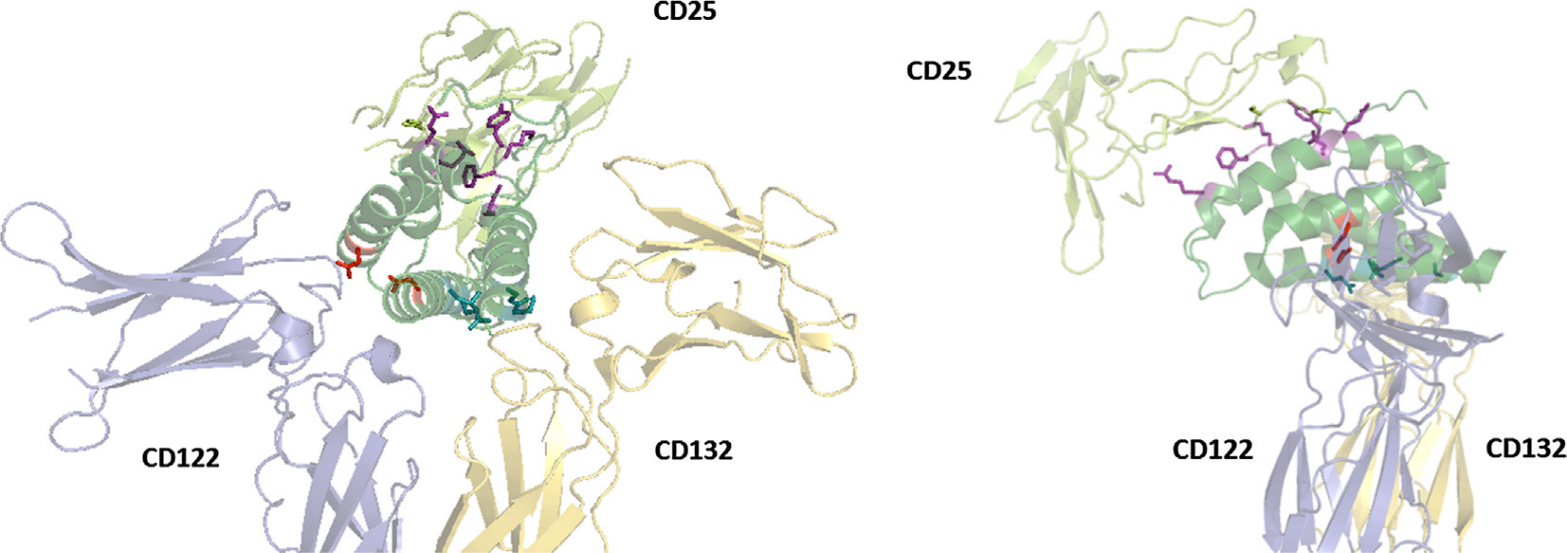
Targeted residues affecting CD122, CD132, and CD25 binding.

Recombinant IL-2 as it is currently approved by the FDA (Proleukin/Aldesleukin) is essentially wild-type IL-2 produced in *e. coli*, and therefore does not possess the O-glycosylation found in human IL2. It also lacks the native N-terminal alanine and a free C-terminal cysteine was mutated to serine (C125S) to reduce disulfide mispairing/aggregation, although this and similar mutations are not expected to affect IL2 function [65], [66]. The most straightforward approach to modifying IL2 function has been to introduce targeted mutations, where even a few specific changes can have profound effects on activity. Mutations introduced into IL2 are typically targeted at sites known to bind CD25, CD122, or CD132 (Fig. 1). This represents an interesting way through which the quality of the subsequent immune response may be manipulated to favor suppressive or cytotoxic responses.

#### 3.1.1. Mutations affecting CD25 binding

Mutations that disrupt CD25 binding are typically employed in order to preferentially direct IL2 stimulation towards cytotoxic CD8+ T and NK cells while limiting interaction with Tregs. Mutation of two amino acids, R38 and F42 were identified to significantly reduce binding to CD25 and the high affinity IL2R. Stimulation of human peripheral blood mononuclear cells with these muteins resulted in lower levels of inflammatory cytokine production in vitro as measured by IL1β, TNFα/β, and IFNγ, and was originally projected to be helpful in reducing IL2 toxicity compared to WT [67]. In order to attain maximal reductions in CD25 binding, multiple mutations have been introduced in combination including the set of R38D, K43E, E61R [68] which targets charged residues, or R38A, F42A, Y45A, and E62A [69], targeting both charged and aromatic residues. IL2 constructs expressing these mutations have demonstrated increased expansion of CD8+ T cells and NK cells and only limited stimulation of T regs in culture as well as *in vivo* models [69].

In contrast, a number of IL2 mutations have also been identified that enhance CD25 binding, including V69A and Q74P [70]. Different combinations of these mutations yielded CD25 binding affinities that could approach 1000 times that of WT IL2 [36], [70]. These high affinity CD25 binders may subsequently serve to enhance IL2R signaling by acting as a cell surface reservoir for IL2 and drive prolonged T cell stimulation and proliferation [36], [71]. In the context of cancer treatment, high affinity CD25 binders may also be useful as T reg antagonists if additional mutations are introduced to disrupt signal activation. This can be accomplished through the introduction of mutations like V91R and Q126T which disrupt IL2 binding at the CD122/CD132 interfaces. Even with WT IL2 binding of CD25, these mutants displayed sub-nanomolar inhibition constants [72].

#### 3.1.2. Mutations affecting CD122 binding

Although most strategies targeting IL2 for cancer currently seek to disrupt CD25 binding as a means to reduce Treg stimulation, mutations affecting CD122 binding have been previously explored as a way to reduce the toxicity of IL2. One amino acid in particular, D20, was proposed to be part of an IL2 “toxin motif (x)D(y)” that resulted in increased toxicity towards endothelial cells [73]. However, D20 is in itself important for CD122 binding [74] and its mutation (eg. D20T/N) leads to reduced proliferation of NK cells and cytotoxic CD8+ T cells without CD3 stimulation [75] due to reduced binding to the intermediate affinity IL2R. Because CD25 binding remains intact, these muteins can still signal through the high affinity IL2R. In this way, some mutations affecting CD122 binding are actually directing towards cells expressing high-affinity receptors, since CD25 binding is now required for signaling. This concept was the basis for an earlier anti-cancer IL2 molecule, named BAY50-4798 (Bayer) containing the N88R mutation which displays preferential binding to high affinity IL2R-expressing cells by virtue of its lack of binding to CD122 alone. Overall, it showed ∼3000 fold greater affinity for the high affinity versus the intermediate affinity IL2R primarily expressed by NK cells. Upon *in vivo* administration, anti-tumor efficacy was comparable to that of WT IL2 upon enumeration of metastases in the B16-F10 model [76]. However, in a phase I clinical trial for metastatic renal cell carcinoma and metastatic melanoma, toxicities qualitatively similar to aldesleukin were observed even in the setting of preferential expansion of T cell subsets over NK cells. This suggests that expansion of NK cells by IL2 therapy is not solely responsible for toxicity *in vivo*. Furthermore, efficacy was limited to only 2 partial responses out of a total 45 patients (ORR < 5%) with the authors noting its insufficient anti-tumor activity for its continued evaluation in this role [77].

Most strategies targeting the IL2-CD25 interaction do so by altering residues directly involved at the binding interface. In contrast, Levin et al. discovered that by changing a series of amino acids mostly situated outside the CD122 binding interface (Q74H, L80F, R81D, L85V, I92F), the conformation of IL2 changed sufficiently to more closely resemble its high affinity receptor-bound state. This resulted in a molecule that could bind to CD122 with ∼200 fold higher affinity, and that could stimulate intermediate affinity receptors with 10x higher potency than WT IL2. When used *in vivo*, the CD122-directed H9 cytokine favored stimulation of CD8+ T cells and NK cells over Tregs and led to less toxicity and greater anti-tumor efficacy in the B16-F10, MC38, and LLC murine tumor models [78].

#### 3.1.3. Mutations affecting CD132 binding

In addition to the inhibitory mutations described by Liu [72], Mitra et al. targeted IL2 binding to CD132 as a strategy to regulate IL2R agonism. They found that by using the H9 IL2 as a base (has high CD122 affinity), they could effectively tune the strength of IL2 signaling by introducing a limited number of mutations at the IL2-CD132 interface, consisting of L18R, Q22E, Q126T, and S130R. In this way, the number of mutations introduced (anywhere from a single mutation to all 4) would inversely correlate with signaling strength, from full inhibition to partial agonism as measured by T cell proliferation, STAT5 phosphorylation, and changes in global gene expression [79]. Mutations in CD132 may represent a complementary way to more finely regulate the overall degree of IL2R signaling.

#### 3.1.4. Potential immunogenicity of IL2 biologics

As with the administration of all biologics, IL2-based therapies bear the risk of immunogenicity and the formation of anti-drug antibodies. Many factors can contribute to the development of these responses including changes in amino acid sequence, alterations in glycosylation, propensity for aggregation, formulation, and even route of administration [80]. The majority of antibodies induced against aldesleukin have been found to be non-neutralizing in nature [81], [82], and although not directly inhibitory, non-neutralizing antibodies may still have the potential to affect drug pharmacokinetics via clearance mechanisms [83]. With a significant number of patients developing antibodies against aldesleukin over the course of treatment [81], additional mutagenesis of IL2 may need to be further evaluated for neo-epitope generation and development of neutralizing antibodies. Screening, identification, and subsequent de-immunization of potentially immunogenic epitopes may therefore be a critically important step affecting the clinical utility of a biologic. An example of successful de-immunization of a cytokine is that of recombinant human IFNβ1, which was demonstrated in a BALB/cByJ mouse model. This was accomplished through the introduction of a single amino acid change (I129V) in an immunodominant T cell epitope identified to be common to humans and BALB/cByJ mice and allowed for retained functionality of the protein while eliminating immunogenicity [84]. This may ultimately serve as a useful approach for other cytokines like IL2**.**

Some residues that have been reported to be important in binding interactions between IL2 and the IL2R signaling complex are shown in Fig. 1. Residues affecting CD122 binding (eg. D20, N88) are shown in red. Residues affecting CD132 binding (eg. L18, Q22, Q126, S130) are shown in teal. Residues affecting CD25 binding (eg. R38, F42, K43, Y45, E61, E62) are shown in purple.

### 3.2. IL2 antibody complexes and IL2 fusion proteins

Instead of introducing mutations to IL2, it is also possible to complex IL2 with anti-IL2 antibodies that occlude CD25 or CD122 binding to achieve analogous effects. Boyman et al. showed that the mouse IL2 antibody clone S4B6, bound IL2 such that interactions with CD25 were disrupted. This allowed for preferential stimulation of the intermediate affinity IL2R but not the high affinity IL2R and led to increased proliferation of cytotoxic cells over T regs. Another antibody clone, JES6-1A12, had the opposite effect, where binding to IL2 led to little proliferation of CD8+ T and NK cells but allowed expansion of T regulatory cells, which was predicted to occur through occlusion of CD122 but not CD25 [85]. This was subsequently confirmed upon solving of the co-crystal structures of S4B6 and JES6-1A12 with IL2, demonstrating overlap of the S4B6 binding site with that of CD25 in addition to allosteric stabilization of CD122 binding. JES6-1A12 occluded binding by CD122/CD132 and induced an allosteric change in the CD25 binding region of IL2 such that CD25 affinity was decreased. However, the IL2 molecule may still bind to cells with high levels of CD25 expression (eg. Tregs). Upon JES6-1A12 dissociation, subsequent signaling can occur through CD122/CD132 and is thought to feed back into a mechanism involving CD25 upregulation [86]. S4B6-IL2 immunocomplexes are indeed found to be more effective at inhibiting tumor growth in B16-F10 [87], [88] and BCL1 *in vivo* tumor models when compared to free IL2 [89] and likely functions through both its cytotoxic cell-directing effects/CD25 disruption and the extension of IL2 half-life to increase biological activity [90].

Administration of IL2/IL2 antibody complexes may also address the issue of vascular leak by reducing the required dose of IL2 to achieve anti-tumor effects as demonstrated in preclinical models [88]. Here, IL2/S4B6 antibody complexes showed superior control of B16-F10 tumor growth at about 40 times less IL2 being administered (as an antibody complex) compared to the high dose group. They further showed that disruption of CD25 function via genetic knockout or antibody inhibition/cell depletion significantly reduced VLS in a C57Bl/6 model. This indicates that therapeutic approaches disrupting CD25 binding (eg. S4B6 or NARA1/IL2 complexes) may not only preferentially stimulate cytotoxic cell types, but also help to reduce vascular leak caused by IL2 stimulation of endothelial cells [88].

Subsequently, the Boyman lab in conjunction with Novartis developed the NARA-1 “mimobody”, an antibody that binds to the CD25-interacting region on human IL2 and functions similarly to the S4B6 antibody in mice [91]. The NARA-1 antibody binds to key residues also involved in the interaction of CD25 with IL2 but with a 10-fold higher affinity (approximately 1 nM). This induces a conformational change in IL2 that is reminiscent of the CD122-directing D10 IL2 molecule [78], thereby also increasing its affinity for the intermediate affinity IL2R. These complexes were successful in mediating anti-tumor effects and increased CD8+ T cell activity in both the B16-F10 tumor model, as well as a spontaneous murine model of melanoma *in vivo* [91].

While shown to be effective in many preclinical models, there may be some potential drawbacks to antibody-cytokine complexes, such as the requirement for pre-complexing of IL2 and antibody prior to administration and the uncertain stability of these complexes *in vivo*. Direct fusion of IL2 to CD25 is another option to favor stimulation of the intermediate affinity IL2R, while sterically obstructing interaction with CD25 on the cell surface. One molecule in clinical trials, ALKS 4230 (Alkermes) [92], utilizes this strategy via creation of a circularly permuted IL2 fused to CD25. Superiority to WT IL2 in syngeneic murine tumor models with further increases in efficacy when combined with checkpoint inhibitors including CTLA4 and PD1 has been presented, but published details are not yet available [93]. In this case, circular permutation may be helpful in achieving optimal activity of an IL2-CD25 fusion protein as fusion of the two molecules directly using a glycine/serine linker may hinder cytokine activity, and subsequently require additional proteolytic processing to achieve full stimulation [94]. Additional information on their molecule/clinical trial is unavailable at this time.

IL2 mutant-Fc fusion proteins may achieve similar NK/CD8+ T cell-directing effects with the added benefit of increased serum half-life through FcRn interactions. Comparison of the fusion of WT versus reduced CD25-binding (R38D, E61R, K43E) IL2 to Fcγ revealed that modification of FcγR function could have unexpected effects on T cell subset depletion and tumor growth [69]. In this case, fusion of the IL2 mutant selectively expanded cytotoxic CD8+ T and NK cells but was actually less effective overall in controlling tumor growth *in vivo* in the B16-F10 model and CT26 syngeneic tumor models as compared to the WT IL2-Fc fusion. WT IL2 Fc fusion anti-tumor efficacy was dependent on FcγR binding and the presence of effector function in order to deplete Tregs from the circulation, as elimination of effector function also decreased efficacy in tumor models. This result implicates a role for Treg depletion in combination with cytotoxic cell stimulation by IL2 in order to achieve superior anti-tumor responses.

It should be noted that although most strategies focus on inhibition of CD25 binding, CD25 interactions may still play an important role in mediating anti-tumor responses. This is exemplified in a study by Su et al. who compared CD122-directing IL2/anti-IL2 antibody complexes with IL15/IL15Ra-Fc complexes for their ability to enhance anti-tumor activity of adoptively transferred CD8 T cells in a B16-F10 model. While IL2/anti-IL2 antibody complexes (S4B6 clone) expanded T regs and IL15/IL15Ra-Fc complexes did not, only IL2/anti-IL2 antibody complexes were successful in enhancing tumor regression when administered with activated CD8 T cells [35]. This is perhaps not necessarily too surprising considering that activated effector T cells upregulate CD25, whereas the interaction of IL15 with CD215 appears to be more important for antigen presenting cells and innate immune responses [95]. However, this does highlight a potential contextual importance for IL2-CD25 interactions, where under basal conditions IL2 may favor T reg stimulation and immunosuppression but under activating T cell conditions (eg. stimulation through TCR/CD3, checkpoint inhibition), IL2 may instead favor stimulation of antigen-specific T cells or NK cells to promote an immune response.

### 3.3. Targeting IL2R-expressing cells (eg. Tregs) for depletion

In contrast to the selective stimulation of cytotoxic cell types the complementary approach of T reg depletion to diminish immunosuppression may also be useful for improving anti-tumor responses [96].

Denileukin diftitox was an early example of a molecule targeting CD25-expressing cancer cells for elimination and was approved by the FDA for the treatment of cutaneous T cell lymphoma in 2008. It consists of amino acids 1-389 of diphtheria toxin (DAB389) fused to human IL2. Upon IL2R binding, the diphtheria toxin is endocytosed and undergoes proteolysis, allowing release of the enzymatically active fragment A into the cytosol. Here, fragment A potently inhibits protein synthesis via ADP ribosylation of eukaryotic elongation factor 2, ultimately inducing cell death [97]. Denileukin diftitox was originally found to be useful in hematologic cancers, in particular cutaneous T cell lymphoma (CTCL), where it could achieve an ORR of 44% [98]. CD25 expression has been correlated with response to treatment where patients with lesions staining for high CD25 expression had clinical responses of 78.5% but patients with low lesional CD25 staining had responses of only 20% [99]. Denileukin diftitox has also been explored as a means of eliminating Tregs in solid tumors. Although it was not initially found to be able to deplete T regs in melanoma patients [100], subsequent studies did show transient reductions in peripheral T regs, sometimes concurrent with other T cell subsets [101], [102], [103]. CD8+ T cell repopulation after depletion subsequently led to the emergence of melanoma-specific T cells and anti-melanoma activity [103]. Denileukin diftitox has been tested in a Phase II trial of metastatic melanoma, where an ORR of 16.7% was achieved only as partial responses [104]. Unfortunately, its current form may function sub-optimally with repeated dosing due to the high frequency of anti-drug immune responses that has developed in patients [105]. Denileukin diftitox was discontinued in 2014.

In place of IL2-toxin fusion proteins, anti-CD25 antibodies may also be employed for Treg depletion, which in this case is dependent on cellular effectors rather than toxin-mediated cell death. Early studies showed that even incomplete Treg depletion via systemic administration of the anti-murine CD25 clone, PC-61, was effective at improving T cell proliferation, IFNγ production, and anti-glioma responses in a dendritic cell-based vaccination strategy [106]. The PC-61 anti-mouse CD25 antibody has little impact on tumor antigen-specific CD25+ cell proliferation *in vivo*, suggesting that the antibody mediates depletion of CD25+ T regs but does not block IL2 stimulation of tumor-reactive effector T cells. Despite this, its inability to deplete intratumoral T regs still represents a major hurdle in the generation of anti-tumor activity [107]. Optimization of the PC-61 isotype from a rat IgG1 to a murine IgG2a resulted in more effective depletion of intratumoral T regs and greater anti-tumor activity in the MCA205, MC38, and CT26 models when used in combination with anti-PD1 [108]. In humans, the CD25 antibody daclizumab was shown to inhibit the suppressive function of CD45RA- Tregs in vitro, rapidly depleted Tregs (∼1 week) in patients with metastatic breast cancer, and did not interfere with tumor vaccine-induced immune responses [109]. However, clinical trials with daclizumab have only shown limited efficacy. One trial in adult T cell leukemia/lymphoma displayed no efficacy in the acute and lymphomatous subtypes (0/18), instead only showing an effect in the chronic and smoldering subtypes as partial responses (6/17, ORR = 37%) [110]. It also did not show efficacy in combination with a dendritic cell vaccine in metastatic melanoma patients [111], where T reg depletion led to the emergence of vaccine-specific T cells but daclizumab also hindered the effector function of these cells [111], though this is in contrast to observations made by Rech et al. [109]. Overall, it appears that T reg depletion may be an important part of an effective anti-tumor treatment combination regimen, but is by itself insufficient to invoke a significant anti-tumor response. Targeting CD25 may also have the unwanted effect of simultaneous depletion/blockade of activated effector cell types and would therefore require careful optimization/scheduling.

A strategy focused on the enhancement of intermediate affinity IL2R activation by saturating CD25 with a high affinity, effector-less antibody has also been described [112]. The antibody was designed to block high affinity IL2R signaling but does not directly induce ADCC of CD25+ cells. In this case, complete blockade of CD25 in conjunction with IL2 stimulation reduced some, but not all indicators of toxicity in mouse models, expanded NK and CD8+ T cells, but also obviated IL2-induced anti-tumor efficacy, which correlated with a loss in tumor-infiltrating CD8+ CD25+ T cells. This suggests that at least with the dosing strategy utilized, CD25-dependent activation of cytotoxic CD8+ T cells was required for anti-tumor efficacy in the B16-F10 and CT26 models (unpublished observations).

### 3.4. IL2 targeting to tumor cells and immune cells

The utility of targeting IL2 to tumor sites was demonstrated by Becker et al. [113] when they fused IL2 to antibodies against different tumor antigens: ch225 with specificity against epidermal growth factor (EGFR, used as a negative targeting control), and ch14.18 with specificity against ganglioside D2 (GD2, used as the antigen of interest). Using this, they were able to better limit B78-D14 tumor metastases in syngeneic models as well as a human melanoma xenograft model treated in conjunction with the adoptive transfer of lymphokine-activated killer cells [113], [114]. A tumor antigen-specific effect in controlling metastases was observed whereby the increase in efficacy was dependent on tumor antigen specificity and was not only an effect of IL2-half-life extension. Despite its efficacy when fused with anti-tumor antibodies, other models have demonstrated the inability of immunocytokines to confine biodistribution to tumor sites as IL2-IL2R binding may dominate over antigen-antibody interactions [115].

Merck has developed several targeted IL2 strategies, of which several have advanced to clinical trials. These include hu14.18 (EMD273063) targeting aberrant GD2 ganglioside expression, huKS (EMD273066) targeting EpCAM, and NHS76 targeting host cell DNA, each fused with either a WT or modified IL2 at the C-terminus of the antibody Fc region. However, these immunocytokines have shown only limited efficacy in patients with metastatic melanoma with a response rate of only 7.1% for hu14.18-IL2 in a phase II trial [116] while huKS-IL2 displayed only stable disease in combination with cyclophosphamide in solid tumors [117]. The immunocytokine NHS76-IL2LT (aka Selectikine, EMD521873) was slightly different from its predecessors in that it was fused to a modified IL2 with the D20T mutation as a strategy to reduce toxicity [75]. This effectively results in an IL2 molecule that, like the N88R mutation of BAY50-4798 [76], [118], decreases affinity for CD122 and should therefore have greater selectivity for cells that express the high affinity IL2R over the intermediate affinity receptor. Phase I trials in patients with solid tumors did not yield any objective responses in combination with radiotherapy or cyclophosphamide [119], [120], [121].

Philogen has also developed an IL2-based immunocytokine (Darleukin) that has been explored in a number of clinical trials. Instead of a full-length antibody, Darleukin is composed of a diabody derived from the L19 antibody which binds the extra-domain B of fibronectin overexpressed in the tumor neovasculature and is fused to IL2 [122]. Although the diabody lacks interaction with FcRn, and hence, has a more limited half-life, its smaller size may also improve its distribution in tissues/tumors [62]. In a phase I/II of solid tumors or metastatic renal cell carcinoma, this molecule showed a short serum half-life 2–3 h. While data from preclinical models were encouraging, no objective responses were ultimately observed, with the best/majority of responses being stable disease [123]. Darleukin has also been paired with other treatments including dacarbazine [124] and L19-TNFα (Fibromun) – an L19 diabody fused to TNFα – in clinical trials for metastatic melanoma [125]. The combination of Darleukin and Fibromun has been tested for efficacy using intralesional dosing, yielding an ORR of 55% with 1 patient experiencing complete remission in all lesions. However, unlike intralesional therapy using only IL2, the combination of intralesional L19-IL2 and L19-TNFα was successful at producing responses at uninjected sites (abscopal effect) although why this is the case is currently unclear [125]. A phase III clinical trial examining the efficacy of L19-IL2 with L19-TNFα administered intralesionally in metastatic melanoma was initiated in 2016 and is ongoing.

Cergutuzumab amunaleukin (Roche) is another immunocytokine that has been engineered to address multiple issues surrounding IL2 therapy. This molecule consists of an antibody against carcinoembryonic antigen (CEA) for tumor localization fused to a mutant IL2 (F42A, Y45A, L72G) to abrogate CD25 binding and enhance cytotoxic cell selectivity. The Fc region of the immunocytokine also includes the mutations P329G, L234A, and L235A which together limit Fc-mediated effector function (ADCC, CDCC) [126]. Potential IL2 toxicity may be further reduced by restricting the number of IL2 molecules to one per immunocytokine, accomplished by creating the antibody in a knob-in-hole format with IL2 fused to the knob chain. Importantly, this molecule was designed in order to achieve a CEA binding affinity higher than that of the modified IL2 for the intermediate affinity IL2R. The goal for this design is to reduce the potential issue of IL2 biodistribution to its receptor and improve localization at tumor sites. Combination with a number of different antibody therapies (trastuzumab, cetuximab, imgatuzumab) universally enhanced efficacies in murine tumor models [127].

Although the majority of studies have attempted to target IL2 to tumor antigens, another interesting strategy has been to instead target IL2 to the desired effector cell type. Ghasemi et al. [128] created a fusion protein for an NKG2D binding protein (OMCP) fused to IL2 with low CD25 binding (R38A, F42K) in order to target NK cells. This molecule demonstrated superior binding as well as activation of NK cells as measured by CD69 and perforin expression without Treg expansion. Its administration improved survival and tumor rejection *in vivo* in the YAC-1 lymphoma and LLC mouse models, demonstrating improved efficacy compared to untargeted IL2.

Importantly, it should be noted that tumor targeting with IL2 immunocytokines may at present, still function sub-optimally. Tzeng et al. [115] demonstrated preferential distribution of a TA99-IL2 immunocytokine to IL2R-expressing innate cells over tumor sites. Although they observed improved efficacy in combination with additional anti-tumor (TA99) antibody, this effect was found to not to be dependent on antigen specificity, as a sm3E-IL2 immunocytokine (a negative control for tumor targeting) produced similar effects in combination with TA99 administration. Instead, improved tumor control was attributed to the longer half-life of the IL2 immunocytokines over recombinant WT IL2. Therefore, traditional immunocytokine approaches using WT IL2 may not necessarily benefit from targeting to tumor sites. Certain approaches to address this issue, including the reduction of IL2 affinity relative to the tumor antigen-specific Fab domains [127], have yielded some improvement in increasing immunocytokine distribution to tumor sites.

### 3.5. Pegylated IL2

The potential benefits to improving the half-life of IL2 were identified early in its clinical development with the emergence of pegylated IL2 in the mid 1980’s [129]. Pegylated IL2 was developed by Cetus (later Chiron) Corporation and consisted of 2–3 PEG chains being conjugated to IL2 via the reactivity of succinimidyl esters to primary amines present in the polypeptide. PEG-IL2 showed greater anti-tumor activity to unconjugated IL2 at equitoxic doses in multiple tumor models and activity correlated with peak IL2 serum concentrations [130]. However, PEG-IL2 did not show increased activity and had similar toxicity to the high-dose IL2 regimen in a phase I clinical trial in metastatic melanoma and renal cell carcinoma. Here, a hybrid dosing scheme to achieve peak serum concentrations with high-dose IL2 was used in conjunction with maintenance doses of PEG-IL2 [131]. More recently, Nektar Pharmaceuticals has developed a releasable PEG strategy where pegylated IL2 is initially administered in an inactive state and gradually gains activity *in vivo*. This molecule is an intermediate affinity IL2R-directing, IL2 prodrug (NKTR-214) consisting of aldesleukin conjugated with six releasable PEG chains [132]. Interestingly, because seven of a total eleven lysines in the mature IL2 polypeptide are located in the vicinity of CD25 interfacial residues, this may help to bias their PEGylation efforts towards the disruption of CD25 binding. In combination with an anti-PD-1 antibody, nivolumab, NKTR-214 achieved a preliminary ORR of approximately 50% in combination with nivolumab in its currently ongoing phase I/II PIVOT trial in patients across a number of cancer types [133], [134]. However, the relative contribution of NKTR-214 to these results may be complicated by the use of PD-1 (treatment) naïve patients who are expected to respond to anti-PD-1 monotherapy (ORR ∼43% from the CheckMate 067 trial in advanced melanoma patients [135]) and the fact that, unlike aldesleukin, NKTR-214 has no efficacy as a monotherapy in solid tumors as observed in their EXCEL trial [133]. The increased tolerability and efficacy of NKTR-214 over early pegylation efforts highlights the importance of the gradual release mechanism of their cytokine *in vivo* to minimize toxicity in addition to its selectivity for cytotoxic cell stimulation which disfavors stimulation of T regs.

### 3.6. Adoptive T cell therapies and chimeric antigen receptors

The field of adoptive T cell therapy shares deep roots with IL2, where peripheral blood cells isolated from cancer patients were initially found to be capable of causing fresh autologous tumor cell lysis with the addition of IL2 [136]. However, it was not until these lymphokine-activated killer (LAK) cells were administered with high-dose IL2 that activity was observed in patients with metastatic cancers [15], [137]. Furthermore, subsequent clinical trials did not demonstrate a significant advantage in the administration of LAKs with IL2 over high-dose IL2 alone in patients with metastatic melanoma or renal cell carcinoma, indicating that the observed anti-tumor effects were primarily mediated by IL2 administration [138], [139]. Despite these initial drawbacks, multiple advancements significantly improved the efficacy of adoptive T cell therapies, including the isolation of tumor infiltrating lymphocytes [140], non-myeloablative lymphodepletion [141], and the development of chimeric antigen receptors [142], [143], [144] that allow for tumor recognition independently of HLA expression and tumor T cell specificity [15]. However, to achieve optimal responses from adoptively transferred cells/CAR-T cells *in vivo*, additional IL2 administration is still needed which may again result in significant toxicity or unwanted expansion of Tregs. As a potential solution, Sockolosky et al. developed an orthogonal IL2-CD122 mutant pair that were specific for each other but not for their wild type counterparts [145]. By introducing two critical mutations into CD122 at the IL2-CD122 interface (H134D and Y135F), they were able to successfully abrogate binding of wild type IL2, and named this mutant CD122 receptor “orthoIL2RB”. This was used to identify specific binding partners using a library of IL2 mutants that did not interact with WT CD122, eventually arriving at a number of compensatory mutations at Q30, M33, D34, Q36, and E37 that allowed for binding of this “orthoIL2” to the orthoIL2RB receptor. Although there was residual binding to WT IL2R at increased doses, orthoIL2 administration was mostly specific for orthoIL2RB transduced lymphocytes *in vivo* and was capable of inducing anti-tumor responses in a B16-F10 model.

Kagoya et al. [146] developed another solution by encoding a truncated portion of the cytoplasmic domain of CD122 in conjunction with a STAT3 binding motif into the CAR itself, potentially obviating the need for exogenous supplementation of IL2. This generated longer lived CAR T cells with higher cytotoxic potential and improved overall survival when compared to those expressing a CD28 or 4-1BB costimulatory domain alone in NALM-6 leukemia and A375 melanoma models. However, it should be noted that although the CD122 cytoplasmic domain was used, examination of gene expression via microarray and gene set enrichment analysis showed signatures analogous to IL-21 stimulation, but not IL-2, IL-7, or IL-15, perhaps due to a dominant STAT3 effect.

## 4. Concluding remarks

Significant progress in the elucidation of IL2 structure and biology has driven the development of unique engineering solutions and more clinically amenable IL2 molecules for use in cancer therapy. Creative strategies to surmount the high toxicities, short half-life, and pleiotropic effects of IL-2 continue to be developed including novel mutational, pegylation, and tumor targeting approaches. Further insights into IL2 structure and biology have yielded additional considerations for the optimization of IL2-based therapies including the potential role for intratumoral T reg depletion in improving responses and the use of modified cytokine-receptor pairs in CAR T cells to reduce toxicity. With the ever increasing repertoire of viable tumor target antigens, the discovery of novel immuno-modulators, and the promise of CAR-T cells, IL2 will likely have an increasingly important role as an effective complement to these immunotherapies in the future.

## Declaration of interest statement

Anthony Tang and Fiona Harding are both employees of and hold shares in AbbVie.
